# The Repeatability of Axial Length Measurements Using a Scheimpflug-based System

**DOI:** 10.18502/jovr.v18i4.14551

**Published:** 2023-11-30

**Authors:** Sara Sardari, Mehdi Khabazkhoob, Ebrahim Jafarzadehpur, Akbar Fotouhi

**Affiliations:** ^1^Research and Technology Deputy, Tehran University of Medical Sciences, Tehran, Iran; ^2^Noor Ophthalmology Research Center, Noor Eye Hospital, Tehran, Iran; ^3^Department of Basic Sciences, School of Nursing and Midwifery, Shahid Beheshti University of Medical Sciences, Tehran, Iran; ^4^Noor Research Center for Ophthalmic Epidemiology, Noor Eye Hospital, Tehran, Iran; ^5^Department of Epidemiology and Biostatistics, School of Public Health, Tehran University of Medical Sciences, Tehran, Iran

**Keywords:** Axial Length, Signal-To-Noise Ratio, Repeatability, Scheimpflug Imaging, Partial Coherence Interferometry

## Abstract

**Purpose:**

To assess the repeatability of Pentacam AXL as a Scheimpflug-based system or measuring axial length according to the age, sex, lens type, axial length value, and type of cataract.

**Methods:**

The present study was conducted using multistage cluster sampling in Tehran, Iran. Ocular biometry was performed, using the Pentacam AXL, by an experienced optometrist on all the participants. The axial length (AL) measurements were taken thrice, with a gap of 10 minutes. To evaluate the repeatability, the intraclass correlation coefficient (ICC) and the repeatability coefficient (RC) were calculated. To determine the significant difference in the repeatability index among study variables, the tolerance index (TI) was calculated.

**Results:**

In this report, 897 eyes of 677 individuals aged between 20 and 91 years (mean ± SD: 64.90 ± 13.62 years) were reported. The ICC of the axial length measurements was 0.981 for all cases. Based on the within-subject standard deviation, the RC was 0.401. The ICC was 0.976 and 0.985 in men and women, respectively. The TI showed better RC of measurements among females. The ICC decreased from 0.999 in participants under 40 years to 0.973 in individuals over 60 years of age. The TI showed a decrease in RC with advancing age. The RC was worse in eyes with nuclear cataracts; the RC was also worse in the first quartile of the signal-to-noise ratio (SNR) compared to the other SNR quartiles.

**Conclusion:**

The Scheimpflug-based systemPentacam AXL had high repeatability in measuring axial length. Some variables such as male gender, older age, and nuclear cataract were associated with reduced repeatability of the measurements. A higher SNR was associated with better repeatability of the axial length measurements.

##  INTRODUCTION 

Axial length is an important ocular biometric component that plays a significant role in determining the refractive status of the eye. Accurate measurement of this index is very important in selecting some ophthalmological interventions. The most important applications of the axial length measurement are to calculate the intraocular lens (IOL) power before cataract surgery as well as to assist in the management of pathological myopia in children. Some reports have shown that more than half of the residual refractive errors after cataract surgery are attributed to errors in the initial axial length calculation.^[1]^ The axial length can be measured with ultrasound or optical biometry methods, each of which has its own advantages and disadvantages. Some devices that are known to be reliable for ocular biometry before cataract surgery include the IOL Master (partial coherence interferometry), the Lenstar LS 900 (low-coherence reflectometry), and the Galilei G6 (Scheimpflug tomography).^[2]^ According to previous reports, the IOL Master is known as the gold standard among the various optical biometry devices.^[3,4]^ In recent years, the agreement of new methods and instruments with the IOL Master has been investigated.^[5,6,7,8,9,10,11,12,13]^ Some studies have shown that the use of new biometric devices due to their high levels of accuracy and more advanced technology has resulted in a significant reduction in the prevalence of residual refractive error after cataract surgery attributable to incorrect measurement of the axial length, from 36% to 17%.^[14,15]^

Pentacam AXL was introduced in 2015. In addition to the features of the previous version (Pentacam HR), this device also measures the axial length using the partial coherence interferometry technique (PCI).^[3,10]^ Shajari et al ^[10]^ conducted the first study on establishing the agreement of the Pentacam AXL with the IOL Master, while the first study on the repeatability of the Pentacam AXL was conducted by Ruiz-Mesa et al.^[16]^ After these reports, a few other studies examined the validity and reliability of the Pentacum AXL.^[17,18,19,20]^ However, it is not possible to generalize the results of existing studies due to their limited number, small sample size, and lack of Pentacam AXL biometric data in eyes with different conditions. Since Pentacam AXL provides tomographic information from the anterior and posterior corneal surfaces, it seems that using this device for ocular biometry can provide more valuable information to surgeons, especially in challenging cases such as keratoconus, post-refractive surgery, and pediatrics.^[21,22,23]^ These new generation formulas used for intraocular lens power calculation provide more biometric and tomographic information.^[24]^ However, because the repeatability of any device is a prerequisite for its validity,^[25]^ it is necessary to examine the repeatability of the Pentacam AXL in different ocular conditions.

Some demographic factors such as age and sex, in addition to directly affecting the AL, also affect the measurement through fixation.^[26,27]^ Moreover, other factors such as crystalline lens status and even the size of the AL may influence AL measurement.^[28,29]^

It should be noted that the repeatability of the Pentacam's axial length measurements has not been addressed according to these variables in previous studies. Therefore, the present report aimed to determine the repeatability of the Pentacam AXL in measuring axial length based on age, sex, type of lens, axial length, and type of cataract.

##  METHODS

The present cross-sectional study was conducted in 2019. The sampling was performed in Tehran, and the samples were selected from all districts of Tehran using the multistage stratified cluster sampling method. Within a predetermined day, all samples were transferred to the examination site free of charge. Complete demographic, socioeconomic, and anthropometric information was first collected by a trained person through an interview, and then optometric and ophthalmological examinations were performed. The examinations included the measurement of uncorrected visual acuity (UCVA), best-corrected visual acuity (BCVA), and presenting visual acuity (PVA) using a LED visual acuity chart (Smart LC 13, Medizs Inc., Korea) at 6 m, auto-refraction by the Nidek ARK-510A auto-refractometer/keratometer (Nidek Co. LTD, Aichi, Japan), ocular health examination of the anterior and posterior ocular segments using the slit-lamp biomicroscope (Haag-Streit AG, Bern, Switzerland) and +90 diopter lens. All participants then underwent optical coherence tomography (OCT) imaging. The exclusion criteria included severe corneal opacity preventing imaging, macular degeneration causing fixation loss, post-vitrectomy eyes, and a history of any intraocular surgery. In the next step, axial length measurements were performed by the Pentacam AXL. In addition to the Pentacam HR properties, the AXL version also measures the axial length using partial coherence interferometry. The biometry was performed by an experienced examiner and three measurements were taken 10 min apart. For some participants, the measurements were repeated four times. Only data that had a signal-to-noise ratio (SNR) greater than 6.3, which is the default for the Pentacam AXL, was recorded. In this study, the classification of cataracts was performed according to the WHO grading system. Nuclear, cortical, and posterior sub-capsular (PCS) cataracts were defined based on the lens opacities of grade 2 or more. Axial lengths of 22 mm or less, 22 to 24 mm, and lengths above 24 mm were defined as short, medium, and long, respectively.

### Statistical Analysis 

Data was analyzed by the SPSS software. Mean and standard deviations (SD) of three and four axial length measurements were reported. The intraclass correlation coefficient (ICC) was calculated to evaluate the reproducibility of the Pentacam AXL's axial length measurements. The repeatability coefficient (RC) was reported by calculating the within-subject standard deviation and multiplying it by 2.77.^[30]^ The closer the RC is to 0, the better the repeatability. The coefficient of variation (CV) was also reported. To examine the statistical difference between the repeatability indices, the tolerance index (TI) was calculated and reported.^[31]^ The tolerance index is the logarithm of the repeatability indices of the two groups relative to each other. For example, if Rm is the repeatability index in men and Rf is the repeatability index in women, the tolerance index will be equal to TI = log (Rm/Rf). To evaluate the significance level of the tolerance index, a significance cut point was obtained based on the sample size in the compared groups according to Bergin et al's report.^[31]^ If the tolerance index was higher than this cut-point, the repeatability index difference was considered statistically significant at a 0.05 α level. The mean and standard deviation of SNR were also reported and its mean was evaluated according to the studied variables by the T-test and analysis of variance (ANOVA). Finally, the first to fourth quartiles of the SNR were calculated and the repeatability index was reported separately for each quartile. The repeatability index was compared between different SNR quartiles by using the calculated tolerance index.

The Ethics Committee of Tehran University of Medical Sciences approved the study protocol, which was conducted in accordance with the tenets of the Helsinki Declaration. All participants signed a written informed consent (Ethics code: IR.TUMS.FARABIH.REC.1399.010).

**Figure 1:**
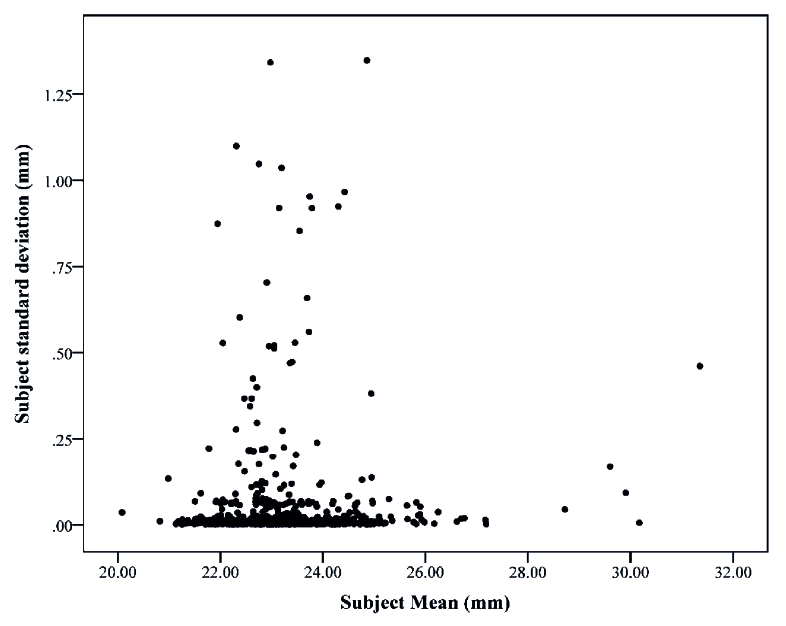
Individual subjects standard deviations plotted against their mean.

**Figure 2:**
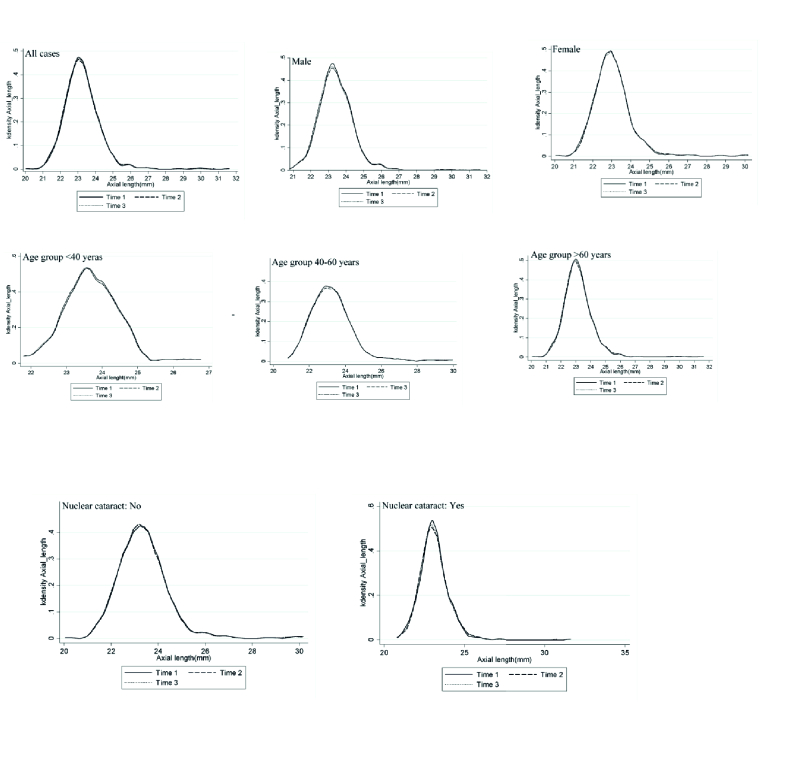
Kernel density plots of axial length in three measurements according to sex, age, and nuclear cataract.

**Figure 3:**
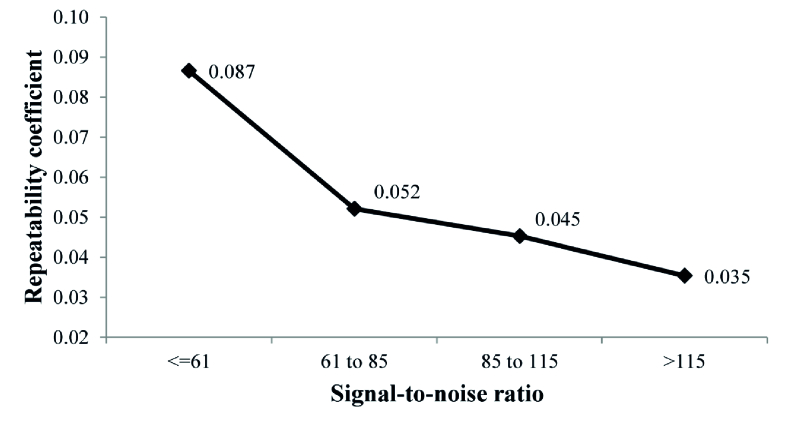
Repeatability coefficient of three time's axial length measurement according to quartile of signal-to-noise ratio (SNR).

**Table 1:** The mean and standard deviation (SD) of axial length in three and four measurements and repeatability indices.


	orangeSNR	orangeTime 1(*n* = 897)	orangeTime 2(*n* = 897)	orangeTime 3(*n *= 897)	orangeTime 4(*n* = 317)	orangeThree measurements			orangeFour measurements			
	orangeMean ± SD	orangeMean ± SD	orangeMean ± SD	orangeMean ± SD	orangeMean ± SD	orangeICC ${}_{3}$	orangeRC ${}_{3}$	orangeCV ${}_{3}$	orangeICC ${}_{4}$	orangeRC ${}_{4}$	orangeCV ${}_{4}$	orangeTI (cut off TI)
	Total	90.90 ± 43.13	23.25 ± 1.06	23.25 ± 1.06	23.23 ± 1.05	23.18 ± 1.09	0.981	0.401	0.18	0.975	0.479	0.25	
Sex	Male	88.17 ± 43.1	23.45 ± 1.03	23.43 ± 1.03	23.41 ± 1.01	23.35 ± 1.1	0.976	0.439	0.20	0.962	0.588	0.33	0.09 (0.06)
	Female	93.66 ± 43.04	23.06 ± 1.05	23.07 ± 1.06	23.06 ± 1.06	22.98 ± 1.04	0.985	0.360	0.16	0.989	0.303	0.17	Reference
Age (years)	< 40	90.38 ± 32.86	23.75 ± 0.85	23.74 ± 0.85	23.73 ± 0.85	23.41 ± 0.76	0.999	0.080	0.06	0.985	0.263	0.20	Reference
	40-60	97.14 ± 42.50	23.29 ± 1.29	23.29 ± 1.29	23.28 ± 1.28	23.07 ± 0.97	0.998	0.160	0.07	0.998	0.122	0.08	0.30 (0.16)
	> 60	89.72 ± 44.60	23.16 ± 1.01	23.17 ± 1.02	23.15 ± 1.00	23.17 ± 1.13	0.973	0.462	0.22	0.972	0.524	0.28	0.760 (0.09)*
Lens	Phakic	92.10 ± 44.23	23.24 ± 0.97	23.25 ± 0.97	23.23 ± 0.96	23.17 ± 1.08	0.976	0.415	0.17	0.973	0.485	0.26	Reference
	Pseudo phakic	87.42 ± 39.62	23.27 ± 1.29	23.27 ± 1.28	23.26 ± 1.27	23.19 ± 1.12	0.990	0.359	0.21	0.978	0.467	0.25	0.06 (0.09)
Axial length (mm)	≤ 22	100.58 ± 46.13	21.65 ± 0.32	21.67 ± 0.37	21.64 ± 0.33	21.63 ± 0.26	0.895	0.307	0.14	0.995	0.051	0.06	0.11 (0.13)
	22-24	93.21 ± 43.34	23.05 ± 0.53	23.04 ± 0.52	23.03 ± 0.52	23.01 ± 0.54	0.924	0.400	0.19	0.885	0.502	0.28	Reference
	> 24	77.14 ± 37.92	24.78 ± 1.19	24.81 ± 1.16	24.79 ± 1.13	24.68 ± 1.37	0.981	0.443	0.17	0.981	0.507	0.23	0.04 (0.11)
Nuclear cataract	No	92.64 ± 42.22	23.38 ± 1.13	23.37 ± 1.13	23.35 ± 1.12	23.22 ± 1.03	0.990	0.319	0.15	0.973	0.472	0.25	Reference
	Yes	87.23 ± 44.83	23.11 ± 0.95	23.12 ± 0.97	23.10 ± 0.95	23.13 ± 1.16	0.968	0.477	0.21	0.976	0.488	0.26	0.17 (0.12)
Cortical cataract	No	91.41 ± 42.06	23.32 ± 1.12	23.31 ± 1.12	23.3 ± 1.10	23.2 ± 1.12	0.983	0.398	0.18	0.976	0.487	0.25	Reference
	Yes	89.25 ± 46.49	23.04 ± 0.80	23.07 ± 0.82	23.04 ± 0.82	23.09 ± 0.94	0.967	0.412	0.19	0.970	0.449	0.26	0.02 (0.09)
PSC cataract	No	91.68 ± 43.11	23.26 ± 1.07	23.26 ± 1.07	23.24 ± 1.06	23.18 ± 1.11	0.982	0.398	0.18	0.974	0.492	0.26	Reference
	Yes	78.28 ± 41.78	23.10 ± 0.86	23.14 ± 0.84	23.08 ± 0.86	23.12 ± 0.8	0.962	0.449	0.17	0.988	0.237	0.13	0.05(0.16)
	
	
white<bcol>14</ecol>SNR, ignal-to-noise ratio; ICC, intraclass correlation coefficient; RC, repeatability coefficient; CV, coefficient of variation; TI, tolerance index *This group was compared to 40-60 years old

##  RESULTS

After applying the exclusion criteria, 897 eyes of 677 individuals were analyzed in this report. The mean age of the participants was 64.90 ± 13.62 years (20-91 years), and 357 (52.7%) were female. Table 1 presents the mean ± standard deviation of the third and fourth axial length measurements by the Pentacam AXL. Figure 1 shows the correlation between the mean axial length in 3 measurements and the standard deviation of the measurements. The ICC, CV, RI, and TI results are presented in Table 1 in detail. In general, the ICC of 3 and 4 axial length measurements were 0.981 and 0.975, respectively. According to the studied variables, the ICC was higher in women than in men; in the under 40 age group the ICC was higher than the elderly; in pseudo-phakic eyes it was higher than the phakic eyes; in individuals with long axial length the ICC was higher than others; and in participants without cataract it was higher than those with cataract.

The repeatability index was 0.401 and 0.479 in 3 and 4 axial length measurements, respectively. The repeatability index has been shown in Table 1 according to the studied variables for 3 and 4 axial length measurements. Based on the tolerance index, this index was 0.09 considering Rm/Rf. According to the sample size of the two gender groups and based on the calculated TI cut-point (0.06), the repeatability was significantly better in females than males. As seen in Table 1, the repeatability was significantly better in individuals aged under 40 years than in participants aged between 40 and 60 years, and it was better in individuals aged 40-60 years as compared to people over 60 years of age. According to the TI results, the repeatability was not significantly different in multiple values of axial length and lens type. The repeatability was significantly worse in individuals with nuclear cataracts and was not significantly different among other types of cataracts (*P* > 0.05).

Figure 2 shows the distribution of 3 axial length measurements by variables whose repeatability index was statistically significant. Table 1 shows the mean and standard deviations of SNR by different variables. The mean SNR was marginally higher in women than in men (*P* = 0.057). However, no significant difference was observed in SNR among different age groups (*P* = 0.191). The mean SNR decreased with increasing axial length (*P* < 0.001). No significant difference was observed in the mean SNR between phakic and pseudophakic eyes (*P* = 0.157). The SNR was marginally lower in eyes with nuclear cataract (*P* = 0.079) and was also significantly lower in eyes with PSC cataract (*P* = 0.030). Figure 3 shows the repeatability index by different quartiles of SNR. As there were 224 eyes in each group, the significance cut point was considered 0.12. According to the tolerance index, statistically significant differences were observed in the repeatability indices of the second SNR quartile compared to the first quartile (TI = 0.22), the third SNR quartile compared to the first quartile (TI = 0.28), and the fourth SNR quartile compared to the first quartile (TI = 0.39).

##  DISCUSSION

An important feature of the Pentacam AXL over the Pentacum HR is the calculation of the axial length and the power of the intraocular lens.^[10]^ The number of studies comparing the results of the Pentacam AXL with other devices, especially the IOL Master are more than the number of studies that evaluated the repeatability of this device.^[3,10][16][17][19][32]^ It is important to confirm the repeatability of the Pentacam AXL before validating it. Since the introduction of the Pentacam AXL in 2015, there have been reports examining its validity and reliability. Shajari et al^[10]^ conducted the first study to evaluate the Pentacam AXL in measuring biometric components, especially axial length. According to the results of this study, the measured axial length by this device was not significantly different from the IOL master 700 and 500, and the results were comparable. Ruiz-Mesa et al^[16]^ and Sel et al^[3]^, studied the repeatability of the Pentacam AXL. In the present study, we examined the repeatability of this device after 3 and 4 axial length measurements. The present study has strengths and limitations that need to be addressed. The most important strength of this study was the evaluation of the repeatability according to different variables such as age, sex, type of lens, axial length values, and type of cataract. This issue to the best of our knowledge has not been addressed in other studies. However, one important point to consider is that Pentacam AXL has a low SNR for high-density cataracts, and we only analyzed data that was acceptable in terms of SNR.

According to the results of the present study, the overall ICC of the axial length measurements was 0.981, although this index varied according to some variables, especially the age of the participants. Ruiz-Mesa et al^[16]^ reported this index in normal and cataractous individuals equal to 0.953 and 0.989, respectively. Sel et al^[3]^ reported this index at 0.995 in individuals aged between 18 and 64 years of age using Pentacam AXL. Some studies have reported ICC values for axial length measurements of the IOL Master 700 around 100%.^[3,33,34,35]^ However, according to the IOL master measurement method (swept-source OCT), its results are expected to have higher validity and reliability. However, the present, and also other studies, on the axial length measurements of Pentacam AXL showed excellent and acceptable repeatability. It should also be noted that the Pentacam AXL has good reliability in measuring anterior and posterior corneal surfaces in both individuals with normal cornea and patients with corneal disorders such as keratoconus.^[21,22,23]^ This device has proven to be highly reliable in measuring axial length, making it a valuable tool in ophthalmology clinics with a high demand for treatment. In certain scenarios, this device may even be a more cost-effective option for patients. Also, considering that many types of corneal and anterior segment indices are used in the new generation IOL calculation formulas, the use of Pentacam AXL can be useful from this perspective. In addition, it offers the ability to validate and double-check multiple measurements simultaneously.^[24]^

The results of the present study showed better repeatability of the axial length measurements in women than in men based on the TI index. This may be due to the better compliance of women toward instructions during the imaging or biometry examinations. However, age may have also played an important role in this finding. In the present study, the mean age of the women was about five years younger than the men. Since the repeatability decreased with increasing age, this sex-related variance may be due to the different age distribution of men versus women in this study.

According to the findings, increasing age was associated with a decrease in the repeatability of the axial length measurements; the repeatability was significantly worse in individuals over 60 years of age than in participants either aged between 40 to 60 years or < 40 years. Better compliance with the instructions during the examinations by younger ages may be a cause of this finding. However, increasing lens opacity and other ocular and systemic diseases that occur with advancing age may also further explain this age-related decrease in repeatability. In general, the decrease in repeatability of the axial length measurements with age seems to be mainly due to increased fixation errors and unwanted shifts in gaze direction due to cognitive changes or disruption of fine motor skills (such as tremors).^[26]^ These conditions have been shown to significantly affect the axial length measurements while using optical biometry and often result in variability in measurement.

Our results showed that the repeatability of axial length measurements was not significantly different in multiple sizes of axial length (short, medium, and long). This finding suggests that the repeatability of the axial length measurements by the Pentacam AXL does not depend on the size of the axial length. However, some studies have shown worse repeatability of axial length measurements in very short or very long axial length values. Our study lacked statistical significance due to the small sample size selected for the short- and long-axial length groups, which was not sufficient to establish a trend. However, as mentioned above, some individuals in the present study were excluded due to retinal problems and the lack of measurement due to very dense cataracts; this should be considered while interpreting results. Therefore, it can be concluded that the Pentacam AXL measurements are repeatable in all axial length values in individuals without prior retinal problems associated with reduced vision.

The most important application of axial length measurement is before cataract surgery. However, according to the results, the repeatability of axial length measurements in patients with nuclear cataract was worse than in other individuals. Limited studies on other optical biometers have reported poorer repeatability in subjects with PSC cataracts.^[16,35]^ The strength of the present study was to show the relationship between the repeatability of axial length measurements and cataracts according to the type of cataract, while other studies only considered the presence or absence of cataracts. Ruiz-Mesa et al^[16]^ studied the repeatability of axial length measurements using Scheimflug and OLCR systems in patients with and without cataracts. Using the data in the Ruiz-Mesa et al^[16]^ study, we calculated the tolerance index. The results showed that the repeatability in the Ruiz-Mesa et al^[16]^ study was significantly different between the two groups (TI = 0.27, significance cut point = 0.33) based on the Pentacam AXL, while the OCLR system did not show any significant difference in the repeatability index between patients with and without cataracts (TI = 0.17, cut point = 0.270); however, better repeatability of the OLCR system was observed. We expected the worse repeatability of Pentacam in patients with cataracts in the Ruiz-Mesa et al^[16]^ study; however, this was not the case, in contrast, the repeatability was worse in normal individuals. We also expected to observe a similar finding in PSC cases, as the mean SNR in eyes with PSC cataract was much lower than in other eyes. In the present study, the grading of the lens opacity in the nuclear and PSC cataract cases was 2.88 and 2.2, respectively. The significant decrease in repeatability in individuals with nuclear cataract may be due to their denser lens opacity.^[28,29]^ Moreover in this study, the small number of PSC cases may not have revealed any significant relationship between PSC cataract and axial length measurement repeatability.

The SNR is an indicator of the accuracy of axial length measurement by the optical biometry devices.^[29,36]^ A high SNR can help inexperienced operators to select the most accurate calculated axial length.^[29]^ The results of SNR analysis in this study confirmed the role of high SNR in the better repeatability of the axial length measurements. A higher SNR confirms a more repeatable and valid measurement. The better signal compared to the amount of noise returned from the eye to the device indicates a more accurate measurement of the axial length. Although these two parameters are measured separately, they are closely related to each other.

In terms of gender, the SNR was better in women which was accompanied by better repeatability of the measurements in females. Regarding the relationship between age and SNR, we did not observe a significant relationship despite an age-related decrease in repeatability. So, this finding suggests that the reduction in repeatability with age can be due to factors other than SNR value such as fixation errors and unwanted shifts in gaze direction. According to the results, patients with cataract, especially the PSC type had lower SNR compared to other individuals. Regarding the reduction of SNR in cataract cases, this finding seems to be mainly due to the reduction in light penetration and increased light scattering.

In summary, the use of the Scheimpflug-based system Pentacam AXL resulted in high repeatability in measuring axial lengths. However, some variables such as male gender, older age, and nuclear cataract were associated with reduced repeatability of the axial length measurements. A higher SNR was associated with better repeatability of the measurements; therefore, attention to the SNR value during imaging, especially in challenging cases, can help select the most accurate axial length.

##  Financial Support and Sponsorship

This project was supported by the Deputy of Research and Technology of Tehran University of Medical Sciences as a PhD Thesis.

##  Conflict of Interest

None.
